# Adult learning: What nurse educators need to know about mature students

**DOI:** 10.4102/curationis.v38i2.1494

**Published:** 2015-11-20

**Authors:** Cynthia Spies, Ielse Seale, Yvonne Botma

**Affiliations:** 1 School of Nursing, University of the Free State, South Africa

## Abstract

**Background:**

Most nurse educators regard students who enter postgraduate studies as adult learners capable of self-direction and independent learner behaviour. Therefore, a mismatch between the nurse educator’s expectation of adult learners and actual adult learner conduct may result in disappointment and even frustration for both educator and learner.

**Purpose:**

This article is a report of a secondary analysis of data that were collected to explore the high-fidelity simulation learning experiences of a group of postgraduate nursing students. The secondary analysis was done to determine whether adult learners who bring professional knowledge and experience to a postgraduate learning environment displayed adult learner conduct as proposed by educational theorist Malcolm Knowles.

**Method:**

Using a qualitative descriptive research design, data were gathered from 18 postgraduate nursing students who participated in high-fidelity simulation in a nursing school at a higher education institution in South Africa. The nominal group technique was used to collect the students’ ideas about improving their simulation learning experiences. A secondary qualitative analysis of the primary nominal group data was done.

**Findings:**

Data either confirmed or belied adult learner behaviour. Although the findings suggested self-directed and independent learner behaviour, they also revealed behaviour evident of dependence on the educator.

**Conclusion:**

Mature students have well established ways of thinking and doing that may hinder learning. Educators have to support adult learners in developing effective learning techniques in order to maximise the benefits of their experience and knowledge by fostering independence and self-direction.

## Introduction

Students in postgraduate nursing programmes are considered to be adult learners. Whilst adult learners can be classified in many ways, this article will focus on mature students who enter higher education and who are defined by Justice and Dornan (2001:236) as non-traditional students aged 24–64. We use the terms ‘students’, ‘adult learners’ and ‘mature students’ interchangeably as reference to non-traditional students who participate in postgraduate studies. Apart from these students being more mature than most undergraduate students, they commonly share at least four non-traditional attributes: financial independence, full-time employment, having dependants and studying part-time (Kenner & Weinerman 2011:87–88). Professional nurses who engage in further education not only share these attributes; they also bring clinical as well as life experiences to the educational environment. In the opinion of Sarah Gravett (2005:8), the existence of a generic adult learner is a myth. She comments that each learner will bring unique characteristics to the learning environment. These characteristics are based upon aspects such as the students’ accumulated life experiences within a specific socio-cultural environment, previous educational experiences and the reasons why they participate in further education. Whilst we agree with Gravett that adult learners are unique, we contend that the dynamics surrounding the learning process of the mature learner is an aspect of adult learning that deserves attention.

Nurse educators might expect nurses who bring professional knowledge and experience to the learning environment to exhibit the characteristics of adult learners. Over the years, research on adult learning has culminated in several lists of adult learners’ characteristics, depending on the research design or theoretical orientation of the researchers (Gravett 2005:8; Merriam 2001:4). Malcolm Knowles influenced adult education practice when he contrasted pedagogy (the art of helping children to learn) with andragogy (helping adults to learn) (Merriam 2001:5). Knowles used andragogy as a concept to explain the conditions and principles for adult learning which include that adult learners:

1. have independent self-concepts and are thus led by self-directedness;
2. draw on their accumulated reservoir of experience, which has a bearing on learning;
3. have learning needs that are influenced by social roles;
4. are problem-centred and want to apply new knowledge immediately;
5. need to know why they have to learn something before participating in learning; and
6. are motivated to learn by internal rather than external factors (Knowles, Holton & Swanson 2005:64–68).

Although this list is by no means exhaustive, we have selected this set of characteristics for the purposes of our research. The students referred to in this article are a group of adult learners who enrolled for a one-year postgraduate nursing course at a higher education institution in South Africa. They are professional nurses who had more than one year’s clinical experience and who had taken full responsibility and accountability for their nursing care, rendered or not rendered. In addition to prior clinical experience, the students were required to meet a number of clinical hours whilst they were studying and were placed in suitable clinical facilities for 20 hours per week during the academic year. Consequently, they had ample opportunities to construct meaning out of the learning materials through interactions with patients, peers and experts in the clinical environment where they were placed. It was not known whether the participating school of nursing’s mature students who had registered for the postgraduate nursing course demonstrated the typical characteristics of Knowles’ adult learners. The research question therefore was whether mature students, who bring professional knowledge and experience to the learning environment, exhibited the characteristics of adult learners as depicted by Knowles’ adult learning theory.

## Background

The adult learners in our study participated in high-fidelity simulation experiences as one of their learning activities. As a learning strategy, simulation is considered valuable in nursing education because it is associated with enhancing creative thinking and developing and demonstrating high-level problem-solving skills (Bland, Topping & Wood 2011:664, 668). Furthermore, simulation increases knowledge, improves learner confidence (Cant & Cooper 2010:3), develops clinical judgement, which is considered an essential nursing skill (Lasater 2007:496), and allows for interdisciplinary collaboration (Rothgeb 2008:494). Apart from these advantages of simulation, the simulation environment is especially suitable for adult learning (Konia & Yao 2013:76). As a student-centred, active and engaging learning experience, simulation allows adult learners to interact with each other, with the simulation environment, as well as with a computerised simulator that can range from low to high fidelity. High-fidelity simulation is usually conducted in an environment where the human simulator is as lifelike as possible, and the students are physically, intellectually and emotionally involved in caring for the ‘patient’ within an authentic scenario (Paige & Morin 2013:e484). The simulated ‘real-life’ situations allow the adult learner to integrate previous clinical and personal experience with scenario events, often revealing areas that require improvement in the cognitive, affective and/or psychomotor learning domains.

Students are debriefed immediately upon concluding a simulation event. Skilful debriefing is considered to be the most important tool for helping students to learn from their experiences, because learning and processing new information occur during debriefing (Issenberg *et al.* 2005:21). As simulation can be a stressful event for students who have never experienced this type of learning, emotions are usually dealt with first in a non-judgmental, supportive manner (Rudolph *et al.* 2007:361). Thereafter, students reflect on and discuss what went well in the simulation and which aspects need to improve. A discussion of the simulation experience not only serves to identify learning needs, but also satisfies adult learners’ need to learn through dialogue (Collins & Martin 2010:198). In addition, feedback provided by the facilitator helps students to evaluate whether they have attained the set outcomes.

In accordance with the viewpoint that adult learners should become part of the process in order to develop better learning experiences and improve their education (Clapper 2010:e10; Fasokun, Katahoire & Oduaran 2005:23), we invited the group of students described at the beginning of this article to participate in our research. Our initial interest was in the group’s suggestions as to how to improve their simulation learning experiences. However, whereas the data indicated that there was room for improvement regarding simulation learning experiences, a secondary analysis of the existing data revealed unexpected findings regarding adult learner behaviour, hence this report.

### Problem statement

Lecturers have often complained that the students in the postgraduate diploma courses do not prepare for class and want to be ’spoon fed’. Furthermore, the study that this report originates from uses Knowles’ adult learning theory as its theoretical underpinning. Therefore the problem statement of this study was whether or not the mature students registered for the postgraduate nursing course at the participating school of nursing demonstrated the typical characteristics of Knowles’ adult learners.

### Purpose

The secondary data analysis that this article reports on aimed to determine whether mature learners in the postgraduate nursing programme exhibit the characteristics of adult learners as described by Knowles’ andragogy.

### Research design and method

Using a qualitative descriptive research design, data were gathered by means of the nominal group technique (NGT). The NGT was developed to overcome the disadvantages of an unstructured face-to-face interview. For example, more ideas might be generated via the NGT than in a formal group discussion because in NGT every participant has an opportunity to contribute, thus avoiding the likelihood of one person dominating the process (Abdullah & Islam 2011:81). The NGT is cost effective and time efficient, and valuable information that accurately reflects participants’ thoughts can be gathered in a single meeting (Potter, Gordon & Hamer 2004:126, 127).

All 21 students on the course were selected to participate in the nominal group. In an effort to minimise coercion, and to increase trustworthiness, an expert facilitator, who was not involved in the participants’ formal education and who had already conducted more than 10 nominal group interviews, invited all 21 students to join the group meeting. Eighteen students, 17 female and 1 male, participated in the nominal group after they had given written informed consent and were requested to preserve confidentiality. Ethical clearance was obtained from the Ethics Committee of the Faculty of Health Sciences at the local university. The vice-rector: academia and the head of the school of nursing gave their permission for the study to be conducted at the school.

### Data gathering

Data were gathered approximately one month after the participants’ sixth and last simulation and debriefing sessions. During the nominal group meeting, the facilitator requested the participants to ‘Write down suggestions to *improve your learning* during a simulation experience’. The participants individually wrote down what came to mind when considering the request. A round robin collection of suggestions followed until all their suggestions were captured on a flip chart. The facilitator discouraged the duplication of ideas, which reduced the amount of narrative data considerably. The participants were not allowed to comment on the suggestions during the round robin stage; instead, they were afforded an opportunity to clarify concepts after all the ideas had been listed (Abdullah & Islam 2011:83; Varga-Atkins 2011:8). Member checking, as a critical element of ensuring the trustworthiness of narrative data, is inherent to the NGT and was applied during the meeting.

The participants generated 26 suggestions as to how to improve their learning during simulation. After all the suggestions were captured on the flip chart, each individual selected five suggestions that they considered most important. After that, they prioritised and scored their five suggestions in the following way: 5 = most important; 4 = second most important; 3 = third most important; 2 = fourth most important; and 1 = least important. After they had prioritised their suggestions accordingly, each individual displayed their ranking slips on the flip chart next to the appropriate suggestions. Finally, the facilitator tallied all the votes of the captured suggestions and arranged them in order of priority. Thus, the quantitative analysis of the data was derived from the scoring and ranking (prioritising) of the participants’ ideas at the end of the meeting (Potter *et al.* 2004:126).

By adhering to the principles of the NGT, rigour was enhanced through the systematic and self-conscious data gathering, interpretation and communication (Nakkeeran & Zodpey 2012:6).

### Data analysis

To interpret the primary quantitative nominal data, we applied deductive interpretive reasoning during a secondary qualitative analysis (Leech & Onwuegbuzie 2007:561, 564, 596). In accordance with the deductive process and with Knowles’s theory of adult learning in mind, we searched for suitable phrases or words that confirmed or belied the characteristics of adult learners. Through a process of co-interpretation we reached consensus about categorising the suggestions and kept a paper trail, thus enhancing rigour. The interpretation was concluded after we had agreed that our findings could be arranged in three categories, as depicted in Table 1.

**TABLE 1:** Secondary analysis findings.

Categories	Themes	Number of suggestions
Human resources and equipment	Not for the purpose of this article	6
Confirmed adult learner conduct	-	3
Conduct in contrast with expected adult learner behaviour	Dependent learner behaviour Feelings of insecurity and discomfort Lack of time management skills	17

## Findings

In the first of our three categories (Table 1), six suggestions related to human resources and equipment, but since these suggestions did not support the purpose of this article, they were not considered relevant for inclusion here.

In the second category (Table 1), three suggestions confirmed adult learner conduct. Two of these suggestions demonstrated self-directedness, which according to Fasokun *et al.* (2005:24), is recognised as one of the key elements of Knowles’ adult learning theory (all suggestions are reproduced verbatim):

‘Debriefing should be included – it helps to improve the next sessions.’

‘Show recordings of the simulations some time after the simulation – it should be optional to watch the videos.’

The third suggestion in our second category also confirmed adult learner behaviour:

‘Look at the group diversity – some quick and others take longer.’

This suggestion again illustrated that adult students are unique and bring different roles and experiences to the educational environment (Gravett 2005:10–11; Kenner & Weinerman 2011:94).

Whilst we found these suggestions encouraging, the remaining 17 suggestions in our third category revealed a contrast between expected adult learner behaviour and actual mature learner behaviour. We have grouped suggestions in this third category under three themes (Table 1): dependent learner behaviour, feelings of insecurity and discomfort, and lack of time management skills.

### Dependent learner behaviour

The following suggestions revealed dependent learner behaviour:

‘Give enough time to prepare oneself before the time – know the topics a week before the simulation session.’

‘Put the topics of the simulations in the module guide and timetable.’

‘Provide a handout of what is going to happen on the simulation day.’

Apart from apparent non-self-directed conduct, the following suggestions revealed participants’ dependence on the educator and their expectation that the educator should ensure a suitable learning environment:

‘Provide step-by-step guidance during the simulation.’

‘Visit the simulation room and environment beforehand.’

‘Practice session before the simulation to ease tension.’

‘Remove unnecessary equipment and make sure that the necessary equipment is available that will be used in the simulation session.’

‘Equipment and stationery must be more or less like in the real situation.’

### Feelings of insecurity and discomfort

It was evident that students felt insecure and uncomfortable in the learning environment, as indicated by the following suggestions:

‘In the beginning, the cameras must be off.’

‘Spectators [*those in the control room*] must be less – only one lecturer.’

‘Debriefing should be conducted by the lecturer only.’

‘Do not ask about the feelings of the student directly after the session.’

‘Debriefing should be done in the simulation venue to alert the memory.’

‘Each member of the simulation group [*participant*] should know beforehand which role he/she will play and what is expected of each role.’

### Lack of time management skills

Contrary to our expectation that professional nurses would be able to complete activities within the reasonable amount of time allowed during simulation sessions, the following suggestions proved the opposite to be the case:

‘Provide enough time to read and understand the scenario.’

‘More time to complete client records – grace period to complete documents.’

‘Provide more time for the actual simulation session.’

## Discussion

From the suggestions in our second category, it seemed that the participants appreciated the debriefing sessions. Apart from revealing some self-directedness, their suggestions corresponded with the principle that adult learners need immediate and frequent feedback as they progress through learning events (Collins & Martin 2010:199). The participants valued the reflective discussions as it inspired them to work out how to improve their performance in upcoming simulation events. They also demonstrated a need to evaluate performance outcomes when they suggested that they should have access to video recordings of simulation events. Since most learning from simulation events usually takes place during the debriefing, we concluded that the participants’ eagerness to engage in such sessions, and to review recordings of the simulation events, revealed a self-directed desire for deeper learning.

The suggestions in our third category were in contrast to the characteristics of experienced practitioners and rather exemplify beginner practitioners who are rule-bound, slow and hesitant, who lack confidence in making decisions and who are overwhelmed by the uncertainty of a situation (ADEA Commission on Change and Innovation in Dental Education 2006:927). According to Benner (2004 cited in Lyneham, Parkinson & Denholm 2009:2479), experienced nurses need not be given clear directions of ways to proceed with tasks as they will naturally draw from previous knowledge, experience and reflection. Expert practitioners are confident about the decisions they make and have an ability to adapt to changing circumstances. The literature on competent and expert practitioners clearly states that those who are competent are able to apply foundational knowledge in diverse settings and circumstances (Nurse Education Stakeholders 2012:50).

Without clarifying how students were prepared for simulation learning events, their suggestions regarding preparation for simulation might seem appropriate. However, when considering that the students’ study guides contained a detailed timetable indicating the outcome(s) and content to be addressed per contact session as well as the scheduled simulation days, it stands to reason that the scheduled simulations reflected the outcomes that should have been attained during the preceding weeks. Prior to the scheduled simulations, the students were invited numerous times to familiarise themselves with the relevant equipment and the high-fidelity simulators that were to be used during scenarios. Considering the preparatory efforts of the educator, on the one hand, and participants’ suggestions, on the other, we concluded that the participants’ suggestions expressed their lack of intrinsic motivation and consequent weak intention to prepare themselves for learning experiences (Joseph 2013:100). We agree with Phipps, Prieto and Ndinguri (2013:14) that individuals with a strong intention to learn will be motivated to do so and will consequently make an effort during learning activities. The educator was therefore concerned that the students did not make use of the opportunities and facilities available to them and depended instead on the educator.

Gravett (2005:6) mentions that adults who have not engaged in educational activities for a while may experience a lack of confidence in their ability to learn new material. A lack of confidence could be demonstrated in a high degree of dependency on the educator and might account for some participants’ need for facilitator guidance prior to and during simulation events. In addition, some participants might have been socialised to regard themselves as passive recipients of knowledge who expect to be taught by an all-providing educator. Consequently, when the mature students were introduced to active, self-directed learning opportunities, they seemed unprepared to perform on their own (Ǻkerlind & Trevitt 1999:97). Ǻkerlind and Trevitt also mention that the introduction of new technologies or other educational innovations, such as high-fidelity simulation in the case of this study, may evoke at least some resistance from students. Since mature students have well-established attitudes, convictions, thinking patterns and educational experiences, new ways of thinking and doing may render learning a difficult undertaking for some of them (Kenner & Weinerman 2011:90). Nevertheless, educators expect that students who enter a postgraduate programme should at least be competent; and when they exit it they should exhibit the expertise of an expert as depicted in Benner’s ‘from novice to proficient practitioner’ model. The high-fidelity simulation sessions were structured according to well-established simulation and healthcare literature. Hence, we anticipated that the students would demonstrate or otherwise develop confidence as they participated in simulation activities.

In accordance with established simulation practice, all our simulation learning events are videotaped. The recordings are used for educational purposes during debriefing sessions, where students have the opportunity to view and evaluate their own behaviour during the simulation. During a pre-briefing session, the students are made aware that their behaviour will be recorded and that members of the simulation team, including their educator, will be observing them from a control room. Although our participants have been reassured that their behaviour will not be graded but only observed, they expressed apparent discomfort and insecurity, as indicated by their suggestions.

Debriefing sessions were facilitated by one or two educators and immediate feedback was provided after the simulation experience. The participants had the opportunity to reflect on the experience. The participants mentioned that reflection is a constructive activity and aims to encourage participants to think about the simulation experience, their actions, emotions and feelings with the result of adding new meaning and a higher level of understanding (Rudolph *et al.* 2007:365). Even though the participants realised the importance of debriefing, their suggestion that debriefing should be done in the same venue as the simulation implied that debriefing in a separate venue after the experience was a challenge for them.

As mentioned earlier, the simulation environment is authentic and corresponds to a fully equipped hospital ward setting. Simulation scenarios were developed to represent a real clinical case as closely as possible. Although none of our participants had prior experience of simulation, their educator felt that they would at least demonstrate familiarity with the simulation environment over time, especially as real clinical settings were represented. Stock and equipment used in the simulations are similar to what is available in the hospital where most of the students’ work integrated learning occurs.

Some authors describe adult students’ ability to draw from their accumulated experience as one of the hallmarks of adult learning (Fasokun *et al.* 2005:40; Gravett 2005:9). Since mature students usually have extensive experience in their field of study, it is expected that they would use their experience as a resource for learning (Clapper 2010:e8). Given that the participants worked in clinical settings together with other professional nurses, it was anticipated that they would demonstrate experience when placed in situations where teamwork is required. Accordingly, a simulation learning experience usually involves three to five students. During a pre-briefing session, specific roles, which corresponded to participants’ current or previous professional roles, were assigned to each member. Their educator did not take much time to explain the assigned roles because it was assumed that the participants would know how to assume the role of ‘sister-in-charge’ or ‘professional nurse’. Furthermore, simulation creates the opportunity for participants to establish a common base of experience. Hence, it was expected that, as adult learners, they would use these opportunities to pool their resources, and interact with and learn from one another. Even so, their suggestion that each member should know his/her role beforehand led us to conclude that the participants found role identification and teamwork a challenge.

Equally unexpected was the lack of time management skills. Simulation sessions were designed so that the students would be able to reach the outcomes within a reasonable amount of time, which was between 20 and 30 minutes. Although we accept that the average mature adult observes more slowly and needs more time to think and react than the average younger person (Gravett 2005:6), the simulation environment is a ‘replica’ of everyday situations in more than one way. We therefore expected our mature students to perform in the same manner as they would have in the actual clinical area. However, even after being exposed to six scheduled simulations as well as discussions of 45–60 minutes about each experience during debriefing sessions, their suggestions made us question their ability to apply their accumulated expertise in the simulation environment.

In our view, the suggestions mentioned in this third category provide evidence that the participants did not fully display expected adult learner behaviour in a structured adult learning environment.

## Conclusion and recommendation

This article should be of interest to nurse educators at institutions of higher education who have to review how mature students conceive of and approach their learning so that educational support can be provided where necessary. Our findings advocate a deeper understanding of the dynamics surrounding the learning process of the mature learner since a mismatch between educators’ perceptions of mature students and an adult learner’s approach to learning may result in disappointment or even frustration for both parties.

A reconsideration of what to expect from mature students’ approach to learning was inspired by a deductive interpretive analysis of narrative data when the results conveyed unexpected findings regarding adult learner conduct. The reasons why the participants conveyed some behaviour that was contrary to the expected adult learner conduct are not entirely clear. The phenomenon might be traced back to previous school and tertiary education, where students were comfortable with traditional lecture-based, content-orientated teaching models and thus have not developed self-directed learning skills. Thus, we recommend that nurse educators make use of diagnostic assessment tools in an effort to acquire information about mature students’ approach to learning. An example is to administer the Self-Directed Learning Readiness Scale (Guglielmino 2013:9) at the beginning of a course to determine the extent to which an educator should build a climate supportive of self-directed learning.

According to Gravett (2005:14), mature students’ life experiences may sometimes be an obstacle to learning. Since mature students have well-established attitudes, convictions, thinking patterns and educational experiences, new ways of thinking and doing might render learning a difficult undertaking for them (Kenner & Weinerman 2011:90). We encourage educators to explore students’ old and new ideas through collaborative discourse and reflection. Regular conversation and reflection related to learning activities could facilitate the construction of new meaning, which in turn might make the learning experience more worthwhile for the mature learner.

We recommend that nurse educators maximise adult learning by creating learning environments that are student-centred as opposed to teacher-centred. This approach will encourage independence and responsibility for learning, which are closely linked to the development of the necessary self-directed learning skills in adult education (Dunlap & Grabinger 2003:7). Various teaching and learning strategies can be used to promote self-directedness; these include the learning contract (Hughes & Quinn 2013:44), computer-facilitated learning (Ǻkerlind & Trevitt 1999:96), problem-based learning (Lack & Bruce 2014:157) and, as in our case, simulation-based learning (Keskitalo, Ruokamo & Gaba 2014:213).

We need to be reminded that mature students learn in different ways. It would be wise, therefore, when nurse educators approach their teaching task, to create learning environments based on an understanding of what the mature student needs; but, more importantly, to create opportunities in which mature students can develop adult learner conduct.

### Limitations

Although we have attempted to give a detailed description of the processes followed in order for readers to decide whether the findings and methodology of this study can be used in their situations, we acknowledge that our study is confined to a particular context and does not necessarily apply to all adult education environments. Furthermore, it was not the intention of our study to highlight possible cultural differences amongst mature students, and we acknowledge that this issue warrants further research.
